# Alterations in Low-Level Perceptual Networks Related to Clinical Severity in PTSD after an Earthquake: A Resting-State fMRI Study

**DOI:** 10.1371/journal.pone.0096834

**Published:** 2014-05-13

**Authors:** Jing Shang, Su Lui, Yajing Meng, Hongru Zhu, Changjian Qiu, Qiyong Gong, Wei Liao, Wei Zhang

**Affiliations:** 1 Mental Health Center, Department of Psychiatry, West China Hospital of Sichuan University, Chengdu, Sichuan, People’s Republic of China; 2 Huaxi MR Research Center (HMRRC), Department of Radiology, West China Hospital of Sichuan University, Chengdu, Sichuan, People’s Republic of China; 3 Radiology Department of the Second Affiliated Hospital, Wenzhou Medical University, Wenzhou, Zhejiang, People’s Republic of China; 4 Center for Cognition and Brain Disorders (CCBD), Hangzhou Normal University, Hangzhou, Zhejiang, People’s Republic of China; University of Electronic Science and Technology of China, China

## Abstract

**Background:**

Several task-based functional MRI (fMRI) studies have highlighted abnormal activation in specific regions involving the low-level perceptual (auditory, visual, and somato-motor) network in posttraumatic stress disorder (PTSD) patients. However, little is known about whether the functional connectivity of the low-level perceptual and higher-order cognitive (attention, central-execution, and default-mode) networks change in medication-naïve PTSD patients during the resting state.

**Methods:**

We investigated the resting state networks (RSNs) using independent component analysis (ICA) in 18 chronic Wenchuan earthquake-related PTSD patients versus 20 healthy survivors (HSs).

**Results:**

Compared to the HSs, PTSD patients displayed both increased and decreased functional connectivity within the salience network (SN), central executive network (CEN), default mode network (DMN), somato-motor network (SMN), auditory network (AN), and visual network (VN). Furthermore, strengthened connectivity involving the inferior temporal gyrus (ITG) and supplementary motor area (SMA) was negatively correlated with clinical severity in PTSD patients.

**Limitations:**

Given the absence of a healthy control group that never experienced the earthquake, our results cannot be used to compare alterations between the PTSD patients, physically healthy trauma survivors, and healthy controls. In addition, the breathing and heart rates were not monitored in our small sample size of subjects. In future studies, specific task paradigms should be used to reveal perceptual impairments.

**Conclusions:**

These findings suggest that PTSD patients have widespread deficits in both the low-level perceptual and higher-order cognitive networks. Decreased connectivity within the low-level perceptual networks was related to clinical symptoms, which may be associated with traumatic reminders causing attentional bias to negative emotion in response to threatening stimuli and resulting in emotional dysregulation.

## Introduction

Posttraumatic stress disorder (PTSD) is one of the most common psychiatric disorders [1]. The core feature of PTSD is the development of characteristic symptoms following a traumatic event, including a lack of emotional equilibrium [2] and the persistent re-experience of stressors, which is accompanied by continuous physiological hyperarousal [3], sustained avoidance of event reminders and a “numbing” of general responsiveness [4]. Although PTSD has a growing and serious impact on the population, little is known about the mechanisms by which this disorder develops. One hypothesis regarding the development of PTSD is the aberrant organization or dysfunction of distributed neural networks involving a triple network model correlation with cognition, including the salience network (SN), central executive network (CEN), and default mode network (DMN) [5]; this hypothesis is supported by previous studies [6], [7]. However, the findings were inconsistent due to confounding factors, including medication, different sources of trauma, and various analysis methods. Furthermore, several recent task-based functional MRI (fMRI) studies have highlighted an abnormal activation in specific regions involving the low-level perceptual (auditory, visual, somato-motor) network in PTSD patients [8], [9]. However, whether the functional connectivity of low-level perceptual and higher-order cognitive (attention, central-execution, and default-mode) networks changes in medication-naïve PTSD patients during the resting state remains unclear. This issue is important for elucidating the pathogenesis of PTSD because the identification of different levels of brain networks and the reorganization of networks may provide evidence that validates the network impairment hypothesis. This modified model involves regions in the circuits that mediate the “contextualization” of stimuli and supplies a novel way to examine the neuro-pathophysiological mechanisms in PTSD patients.

In recent years, the resting-state fMRI has been identified as a marker of neural connectivity to explore the alterations of coherent intrinsic neuronal activity of blood oxygen level-dependent (BOLD) fluctuations in the brain. ICA is a data-driven analysis that separates a set of signals into independent, uncorrelated, and non-Gaussian spatiotemporal components without requiring a priori specification of a seed region [10]. ICA is particularly valuable for the investigation of brain networks modulated by task performance [11] and at rest [12], [13]. Among them, the auditory network (AN), the visual network (VN), and the sensory-motor network (SMN) are composed of elementary networks that would be of considerable interest because they have connections with the situationally accessible memory system (SAMs). This system is triggered involuntarily by environmental or internal cues that are reminiscent of the original trauma for individuals and form the basis for flashbacks that are re-experienced by the individuals [14].

The primary aims of the present work were to use the task-free resting-state fMRI to investigate the two-level network effects on the pathogenesis of PTSD. Two hypotheses were considered. First, whether the functional connectivity of RSNs related to perceptual and higher cognitive processes may be aberrant in traumatized participants with PTSD. Second, whether any of the changes identified would be related to the measured clinical severity.

## Methods

### 2.1 Subjects

The study was approved by the Medical Research Ethics Committee of West China Hospital, Sichuan University and all the subjects’ written informed consents were obtained before the study. We acquired whole-brain resting-state fMRI for 18 patients (43.33±8.04 years, all right-handed) and 20 non-PTSD individuals with a history of trauma exposure (40.62±9.20 years, all right-handed). All participants were recruited two years after the Wenchuan earthquake through the Mental Health Center of the Huaxi Hospital, Chengdu, China (Table 1). Population age, gender and education characteristics were matched between patient and control groups. All participants were less than 60 years of age and were interviewed to confirm that there was no history of psychiatric illness among their first-degree relatives, no history of head injury or neurologic disorders, and no history of drug or alcohol abuse in the six months preceding the scan. Participants were evaluated with the Clinician-Administered Posttraumatic Stress Disorder Scale (CAPS) [15], which had excellent test-retest reliability across all clients and displayed moderate convergent validity for PTSD [16]; the Hamilton Anxiety Rating Scale (HAMA) and the Hamilton Depression Rating Scale (HAMD) were also used. The diagnosis of PTSD was determined by a consensus between the two attending psychiatrists and a trained interviewer using the Structured Clinical Interview DSM-IV (SCID)–Patients Version. PTSD patients receiving psychotherapy or psychiatric medications and participants with a total CAPS score of less than 40 points were excluded.

**Table 1:** Psychological or behavioral data.

	PTSD (n = 18)	HS (n = 20)	PTSD vs. HS	
	M± SD	M± SD	T value	p value
Gender(n: male/female)	4M/14W	11M/9W	–	0.052^a^
Age (yrs)	43.33±8.04	40.30±9.32	1.07	0.293
Education (yrs)	7.11±3.68	8.65±3.39	−1.34	0.188
CAPS	63.94±13.53	11.35±8.32	14.60	<0.0001
HAMD	13.83±5.39	4.00±3.49	6.59	<0.0001
HAMA	13.94±4.53	3.95±4.12	7.06	<0.0001

Data from questionnaires are presented in terms of mean score (M) and standard deviation (SD) in PTSD and HS groups. Statistical comparisons between the two groups are also provided.

PTSD, posttraumatic stress disorder; HS, healthy survivors; CAPS, Clinician-Administered Posttraumatic Stress Disorder Scale; HAMA, Hamilton Anxiety Rating Scale; HAMD, Hamilton Depression Rating Scale.

a The p value was Fisher’s Exact test. The other p values were obtained by two-sample two-tailed t -test.

### 2.2 Image Acquisition

Experiments were performed on a 3.0-T GE-Signa MRI scanner (EXCITE, General Electric, Milwaukee, WI, USA) with an eight-channel phased array head coil at the Huaxi MR Research Center. We immobilized the participants’ heads with foam padding. Functional images were acquired using a single-shot, gradient-recalled echo-planar imaging sequence (TR = 2,000 ms, TE = 30 ms, and flip angle = 90°). Thirty transverse slices (FOV = 240×240 mm), which had a matrix size of 64×64 and a slice thickness of 5 mm without a gap, yielded a voxel size of 3.75×3.75×5. A total of 200 volumes were acquired and images were aligned along the anterior commissure–posterior commissure (AC–PC) line. Subjects were instructed to relax, let their minds wander, keep their eyes closed, and not fall asleep.

### 2.3 Data Preprocessing

The data were preprocessed using SPM8 software (http://www.fil.ion.ucl.ac.uk/spm). All data were corrected for slice timing, and head motion correction of the functional scans was performed. Next, the functional images were coregistered to the mean functional image and normalized to the echo-planar imaging template in SPM8. The data from 1 of the 19 subjects in the PTSD group were excluded because the translation and rotation of the head motion exceeded ±1.5 mm and ±1.5°, respectively. Images were then spatially smoothed by convolution with a half-maximum isotropic Gaussian filter (FWHM = 8 mm).

### 2.4 ICA and Identification of RSNs

Briefly, ICA is a mathematical procedure to decompose, for example, a spatiotemporal signal into independent, uncorrelated, and non-Gaussian components. We performed a spatial ICA using the GIFT software (http://icatb.sourceforge.net/,version 1.3e) [17]. To determine the number of independent components (ICs), we estimated the dimensions of the datasets from the two groups using the minimum description length (MDL) criterion to account for spatial correlation [18]. FMRI data from all subjects in each group were then concatenated, and the temporal dimension of the aggregate dataset was reduced via principal component analysis (PCA), decomposed by IC estimation (with time courses and spatial maps) using the Informax algorithm [17], [18]. Two separate analyses based on the spatial ICA were conducted on the PTSD and healthy survivor (HS) groups, with 44 and 46 ICs, respectively. The intensity values in each map were scaled to z scores because ICA of fMRI data intrinsically extracts patterns of coherent neuronal responses (i.e., networks) [11], [19]–[21].

### 2.5 Component Identification

Our RSNs were chosen according to the available knowledge on the psychopathology of PTSD and its neuronal correlates. Similar to other anxiety disorders, PTSD is related to difficulty in managing emotions, but particularly with an attentional bias for threatening stimuli [22], and intrusive memories [23]; these factors rely on the SN, CEN, and DMN. Because neurobiological studies of episodic memory retrieval have also shown that the visual cortex and auditory cortex are preferentially activated during reminder exposure tasks [8], [9], we also chose the VN, AN, and SMN. To date, there is no consensus on how to select the optimal number of components, although methods to do so are in development [17]. We used RSN templates provided by previous studies [24], ensuring that the RSNs had a similar spatial pattern in the two groups [18]. The components were selected based on the largest spatial correlation [25], extracted from all subjects, and corresponded to six RSNs in the current work: the SN, DMN, CEN, SMN, VN, and AN.

### 2.6 Second-level Analysis of the RSNs

**Figure 1:**
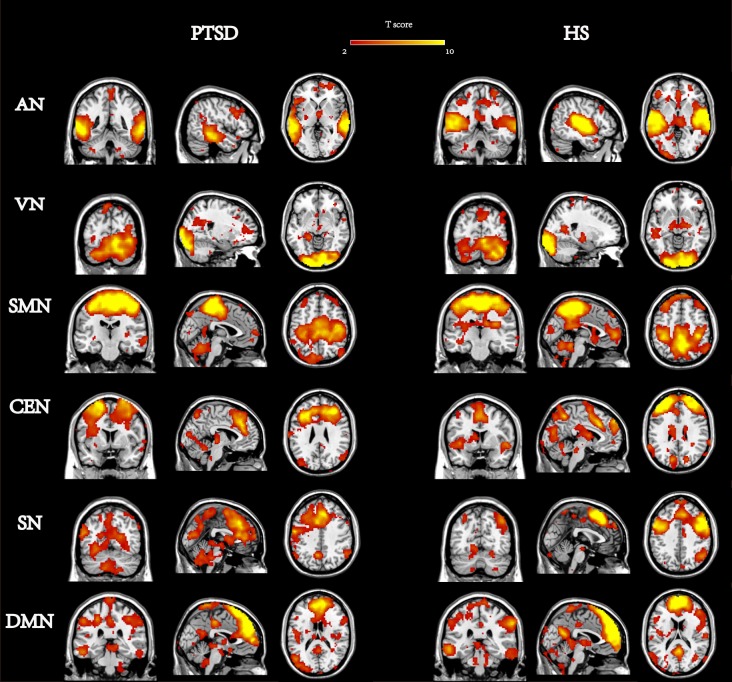
Six networks between PTSD and Healthy survivors (HS). (p<0.05, FDR corrected).

The spatial maps of each RSN were then gathered in each group for a random-effect analysis using one-sample t-tests (Fig. 1); data were corrected using a false discovery rate (FDR) with a threshold of p<0.05 in SPM8. The spatial distribution of the RSNs was the same as in previous studies [24], [26]. To qualitatively and quantitatively compare the RSNs between the PTSD patients and HSs, the resulting maps of two-sample t-tests were thresholded at p<0.05 and corrected for multiple comparisons using a FDR correction [27] with an extent threshold of 10 voxels using the toolbox of REST (http://www.restfmri.net/forum/). The group comparisons were restricted to the voxels within the corresponding RSNs.

### 2.7 Post hoc Correlation Analysis

Pearson correlation analysis was performed between the connectivity strength (mean z-values) and the CAPS, HAMD, and HAMA scores to investigate the relationship between the z-values in the network maps and clinical severity. We extracted the z-values emerging from significant clusters of interest between the PTSD and HS groups as a mask consisting of several regions of interest (ROIs), which were applied to all subjects. The mean z-values of each individual within these ROIs were correlated to the clinical scales and then thresholded at a significance level of p<0.05.

## Results

### 3.1 Psychological and Behavioral Data

Group demographic characteristics and psychological and behavioral scores are shown in Table 1. Compared with the HSs, PTSD patients displayed significantly impaired performance of CAPS, HAMD, and HAMA with higher scores.

### 3.2 Spatial Pattern of RSNs in Each Group

Our analysis indicated that the six RSNs were spatially consistent across patients and controls; the findings were consistent with previous fMRI studies on RSNs [12], [21], [26], [28]. According to the one-sample t-tests, these six RSNs in both the PTSD and HS groups were the SN, CEN, DMN, SMN, AN, and VN (Fig. 1 shows the ICs of the PTSD and HS groups corrected using FDR with a threshold of p<0.05). The SN primarily contains the superior and middle prefrontal cortices, the anterior cingulate and paracingulate gyri, and the ventrolateral prefrontal cortex. The CEN connects the dorsolateral frontal cortex with the parietal cortex. The DMN is composed of the posterior cingulate cortex (PCC), the anterior cingulate cortex (ACC), the middle temporal gyrus, and the medial prefrontal cortex (mPFC). The SMN includes the pre- and post-central gyrus, the primary sensory-motor cortices, and the supplementary motor area (SMA). The AN primarily encompasses the bilateral middle and superior temporal gyrus, the Heschl gyrus, and the temporal pole. The VN is anchored in the inferior, middle, and superior occipital gyrus, the temporal–occipital regions, and along with the superior parietal gyrus.

### 3.3 Aberrant RSNs in Patients with PTSD

**Figure 2:**
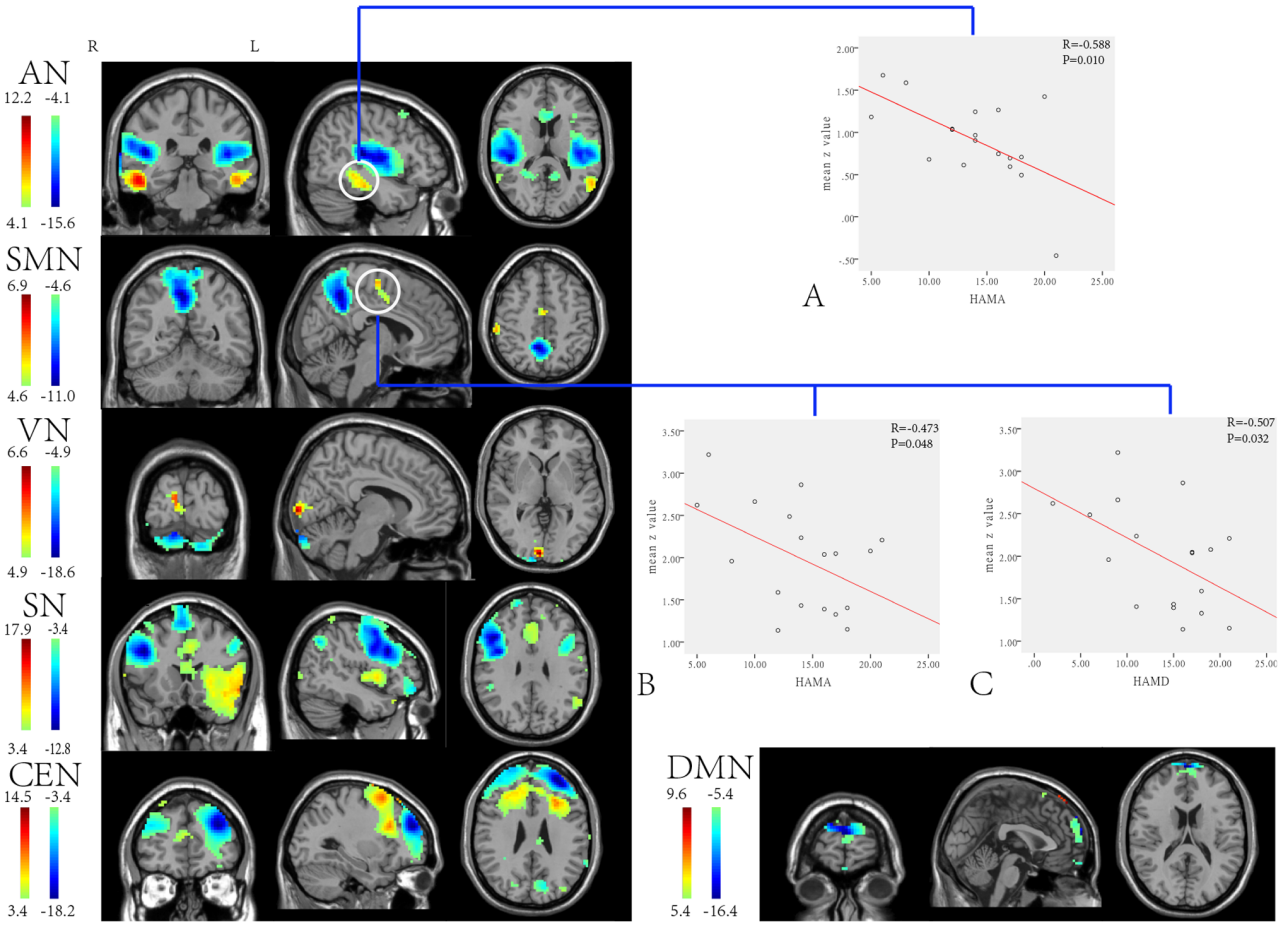
Anatomic templates implement of a two-sample t -test each RSNs in the PTSD vs. HS (p<0.05, FDR corrected). The warm and cold colors indicate the brain regions with significantly increased and decreased functional connectivity in PTSD, respectively. Correlation plots (p<0.05) between functional connectivity and psychometric measures, between mean Z values of ROIs(regionals of interest)and the clinical severity scores in PTSD patients. (A) Correlation between inferior temporal gyrus and HAMA score. (B) Correlation between supplementary motor area(SMA) and HAMA score. (C) Correlation between supplementary motor area(SMA) and HAMD score.

The two-sample t-tests revealed differences in the functional connectivity between the two groups (Fig. 2; Table 2). Direct group contrasts confirmed that both increased and decreased (p<0.05, FDR corrected) functional connectivity were illustrated in the SN, CEN, DMN, AN, VN, and SMN of PTSD patients. Moreover, supplementary HAMA values in the PTSD patients exhibited a significant (p<0.05) negative correlation with the mean z-values of the inferior temporal gyrus (IFG) in the AN. The negative connection strengthened between the mean z-values of the SMA in the SMN and the HAMA values, as well as the mean z-values of the SMA in the SMN and the HAMD values.

**Table 2:** Difference of functional connectivity of the brain regions (PTSD- HS) in each RSN, along with the MNI coordinates of the peak foci and the associated Brodmann areas (BA).

RSN	ROI	BA	cluster	t-value(peak)	MNI		
					x	y	z
SN	Middle frontal gyrus (L)	8	797	−12.82	45	18	30
CEN	Medial frontal gyrus (L)	9	439	−18.23	−30	48	27
DMN	Orbital frontal gyrus (L)	10	64	−16.43	0	66	18
AN	Superior temporal gyrus(R)	22	492	−15.65	45	−30	15
	Inferior temporal gyrus (R)	20	69	8.17	60	−54	30
SMN	Precuneus (R)	31	473	−11.00	6	−54	48
	Supplementary motor area(R)	6	37	5.62	9	−15	60
VN	Cuneus(R)	18	60	6.55	6	−96	0
	Cerebelum(R)	–	159	−18.63	15	−87	−27

BA, Brodmann area; MNI, Montreal Neurological Institute; ROI, region of interest; threshold was set at P<0.05 (FDR corrected).

## Discussion

Consistent with our hypothesis, the current study reveals the different functional connectivities in both low-level perceptual and higher-order cognitive networks in earthquake survivors with and without PTSD. Furthermore, both decreased and increased functional connectivity were found in all six networks in PTSD patients relative to HSs, whereas only changes of low-level perceptual networks were related to clinical severity, as assessed by the HAMA and HAMD scores. We also noticed that PTSD patients displayed significantly higher scores of HAMD, and HAMA when compared with the HSs, because most of PTSD patients are accompanied by physiological anxiety symptoms and negative apprehension. These findings provide further evidence that widespread changes occur in the cognitive networks of PTSD patients; these alterations may be related to stress-related impairments, possible functional reorganization, and/or a compensatory mechanism [29]. Changes to low-level perceptual networks may play a critical role in the pathogenesis of PTSD symptoms.

### 4.1 Low-level Perceptual Networks in PTSD

The most interesting findings of the current study were those of the deficits of the AN and SMN, which were related to clinical severity in these earthquake-related PTSD patients. The AN and SMN are the most important components of the low-level perceptual network; this network can be accessed involuntarily and forms the basis for the flashbacks and nightmares that relate to the traumatic moments in PTSD [30]. These findings support the dual representation theory of the development of PTSD, which was developed by Brewin [31] and in which the SAMs contains detailed sensory and perceptual images and holds low-level representations that are tightly bound to their sensory and affective qualities. In PTSD, extremely stressful events are stored as SAMs that are linked with sensory systems, including the AN, VN, and SMN.

#### 4.1.1 The AN in PTSD

Lesions of the AN are influential for tinnitus, which was one of the most frequently reported problems among veterans returning from two recent armed conflicts, often co-occurring with PTSD [32]. The current study extended the previous findings by indicating complex changes of strengthened interconnection within the AN in PTSD patients relative to HSs, i.e., PTSD patients had both higher connectivity involving the inferior temporal gyrus (IFG)/fusiform and lower connectivity involving the superior temporal gyrus. Downar et al. [33] used event-related fMRI to identify IFG responses to oddball or otherwise salient stimuli and inferred that IFG may play a broad role in evaluating the potential relevance of sensory stimuli and in inhibiting prepotent responses to stimuli. Because PTSD is typically associated with traumatic reminders, elevated functional connectivity of the visual association cortex, such as the IFG reported in previous studies, can be interpreted as representing vivid intrusive memories that occur spontaneously and are accompanied by dissociative distortions to the perception of time, place, and self from even relatively mild stimuli [34], [35]. In addition, the negative correlation between functional connectivity in the IFG and HAMA score provides further evidence for anticipatory anxiety, the discontinuity of conscious experience, and impaired memory retrieval in PTSD patients in the resting state.

The superior temporal gyrus (STG) is involved in nonverbal auditory and language processing and has been found to be activated in dissociated PTSD subjects during recall of the traumatic memory [36]. The disrupted function of the STG observed in patients with PTSD suggests that temporal or limbic structures may contribute to the patients’ dissociative responses, which involve the fragmentation of the typically integrated function of consciousness, memory, identity, or perception of the environment [37]. Alternatively, our findings may be due to the critical role of the STG in social cognition [38], which is connected with the avoidance of necessary cognition and affective processing of trauma [39] that is typical in PTSD. The above data are attributed to the anatomy of the STG, which is a vital structure in the pathway involving the amygdala and the prefrontal cortex that supports social cognition processes and encodes memory involvement [38], [40].

#### 4.1.2 The SMN in PTSD

Another network showing correlations with the severity of clinical symptoms in PTSD was the SMN. The SMN is operationally defined as the region that has significant functional connectivity with the primary somato-motor cortex (pre- and post- central gyrus) [41]. The SMN contributes to motor skill learning, as evidenced in animal models and human motor controls, which is accompanied by changes in the strength of connections within the primary motor cortex [42]–[44]. The current investigation demonstrates that regional connectivity in the SMA is a key part of the ‘salience network’ that processes autonomic, interoceptive, homeostatic, and cognitive information of personal relevance [33], [45]. Thus, we propose here that increased functional connectivity of the motor cortex may represent the neural correlate of preparation for coping with a physical threat [46], in line with the psychobiological mechanisms underlying PTSD. Cunnington et al. [47] have found that the SMA plays a common role in encoding or representing actions prior to voluntary self-initiated movements; the early component of premovement activity is strongly influenced by higher cognitive factors, such as attention. Our findings of a significant negative correlation between the SMA and the HAMA and HAMD factors further indicate that patients with PTSD tend to focus their attention upon and are hypervigilant for information related to ‘emotional alarms’ and associated stimuli. Furthermore, this attentional bias to negative emotion has been evidenced with the Stroop task [48].

The precuneus has been proposed to be involved in cognitive function, such as memory processing and spatial location encoding [49], [50]. A reduced-strength connectivity of the precuneus correlated with other regions typically involved in the default mode network has been observed in PTSD patients during the resting state in previous fMRI studies [51]. This finding suggests the possible involvement of the region in the cognitive deficits seen in PTSD [52], [53]. Given that the process of mental image generation requires reactivation of a stored percept, the precuneus may therefore be important in the retrieval of spatial information [54], which is associated with the core PTSD symptoms related to intrusive thoughts and memories.

#### 4.1.3 The VN in PTSD

Excessive vigilance as a hallmark of PTSD may be associated with increased demands on the brain areas that are involved in the visual association cortex in pathological memories and planning a response to potentially threatening stimuli [46]. Our findings indicate an increased functional connectivity of the cuneus in the VN, which is consistent with previous studies [36]. This increased functional connectivity of the region is likely associated with an ongoing vigilance response [55] and with spontaneous dissociative experiences, including modulations of mental imagery [56]. Patients with cerebellar damage have been found to have reduced cognition and motor capacity in the verbal-visual span-matching task. Therefore, the breakdown in the functional connectivity of the cerebellum seen in our research provide further evidence for the impairment of verbal working memory (VWM) that is typical in PTSD [57].

### 4.2 High-level Cognitive Networks in PTSD

The present findings indicate that the Brodmann 8, 9 and 10 which are the constituents of the SN, CEN, and DMN, respectively, have decreased functional connectivity in PTSD patients when compared to HSs (Fig. 2 and Table 2). These regions belong to an extension of the medial prefrontal cortex (mPFC) [58] and involve the most replicated findings in PTSD, including diminished activation in the mPFC in PTSD patients relative to healthy controls regardless of a history of trauma exposure [5]. Further, these findings are in harmony with the anatomy and function of the mPFC, which was noted through the inhibition of excessive cortico-limbic activity [59]. Given that the mPFC has been shown to activate during self-referential and emotional-processing tasks [60], [61], we speculate that the reduced activation of the mPFC in PTSD may be a reflection of the self-relevant nature of the traumatic stimuli and signify a deficiency in emotional self-awareness during traumatic memory recall [62]. In addition, the above findings support the prominent cognitive behavioral models of PTSD, which include an excess of fear memory of the traumatic event, a failure of expression, derealization states, and dissociative amnesia [63]. Interestingly, we did not identify any activation of the amygdala, which has been a focal point in the high-level cognitive networks; from this, we inferred that there may only be a consistent site of greater activation of the amygdala in comparison to individuals from a non-trauma-exposed group rather than the traumatic exposure control group. These data are in line with the notion that diminished medial prefrontal function is a reliable neural marker of PTSD and makes contribution to trauma-related resilience whereas amygdala may be connected more with arousal experience [5].

## Limitations

Our results have several limitations. First, the absence of a healthy group that never experienced the earthquake limited the investigation of the discrepancy among the three groups. Alterations in cerebral function may be driven by trauma exposure rather than being unique in PTSD [64]. A second limitation is the small sample size considered in this study. Furthermore, we did not use physiological monitoring of the breathing and heart rates, which may interfere with the detection of spontaneously occurring low-frequency ranges of brain activity during the scan. For slow sampling rates (as in this study where one brain volume was scanned every 2 s), structured noise from these two physiological mechanisms can interfere with the low-frequency oscillations at which the resting-state connectivity is detected [65], [66]. The majority of the PTSD patients were women, which is attributed to patient recruitment bias. The sex difference between subjects did not affect the within-study results because the patient and control groups were matched on all variables.

Future research should also use specific task paradigms that may reveal perceptual impairments. Ideally, such an optimized network metric may prove efficacious in making the critical clinical distinction regarding which at-risk subjects will develop PTSD or remain healthy.

## Conclusions

In summary, the results of our baseline study provide a comprehensive examination of the relationship between the elementary and higher cognitive networks in PTSD patients exposed to a single trauma. The lower order of the cognitive processing hierarchy is directly related to symptom severity and contributes to our understanding of the mechanisms involved in emotion regulation and memory recall from trauma in patients with PTSD. Diminished medial prefrontal function as a reliable biomarker of PTSD provides evidence for contextualization impairments, including emotion regulation, social cognition, and self-referential processing [5]. Future studies that are combined with more sophisticated techniques, such as genetics, pupillometry, heart rate variability, and electroencephalographs (EEG), or integrated with task-related activities may yield more refined results about the neurophysiological processes of PTSD.
